# Supplementary material for: LRH-1 agonism favours an immune-islet dialogue which protects against diabetes mellitus
Source: Nat Commun. 2018 Apr 16;9:1488. doi: 10.1038/s41467-018-03943-0 (PMC5902555; doi:10.1038/s41467-018-03943-0)
Supplement: Supplementary file 1 — Supplementary information [file 41467_2018_3943_MOESM1_ESM.pdf]

LRH-1 Agonism Favours an Immune-Islet Dialogue which Protects Against Diabetes Mellitus

Cobo-Vuilleumier *et al*.

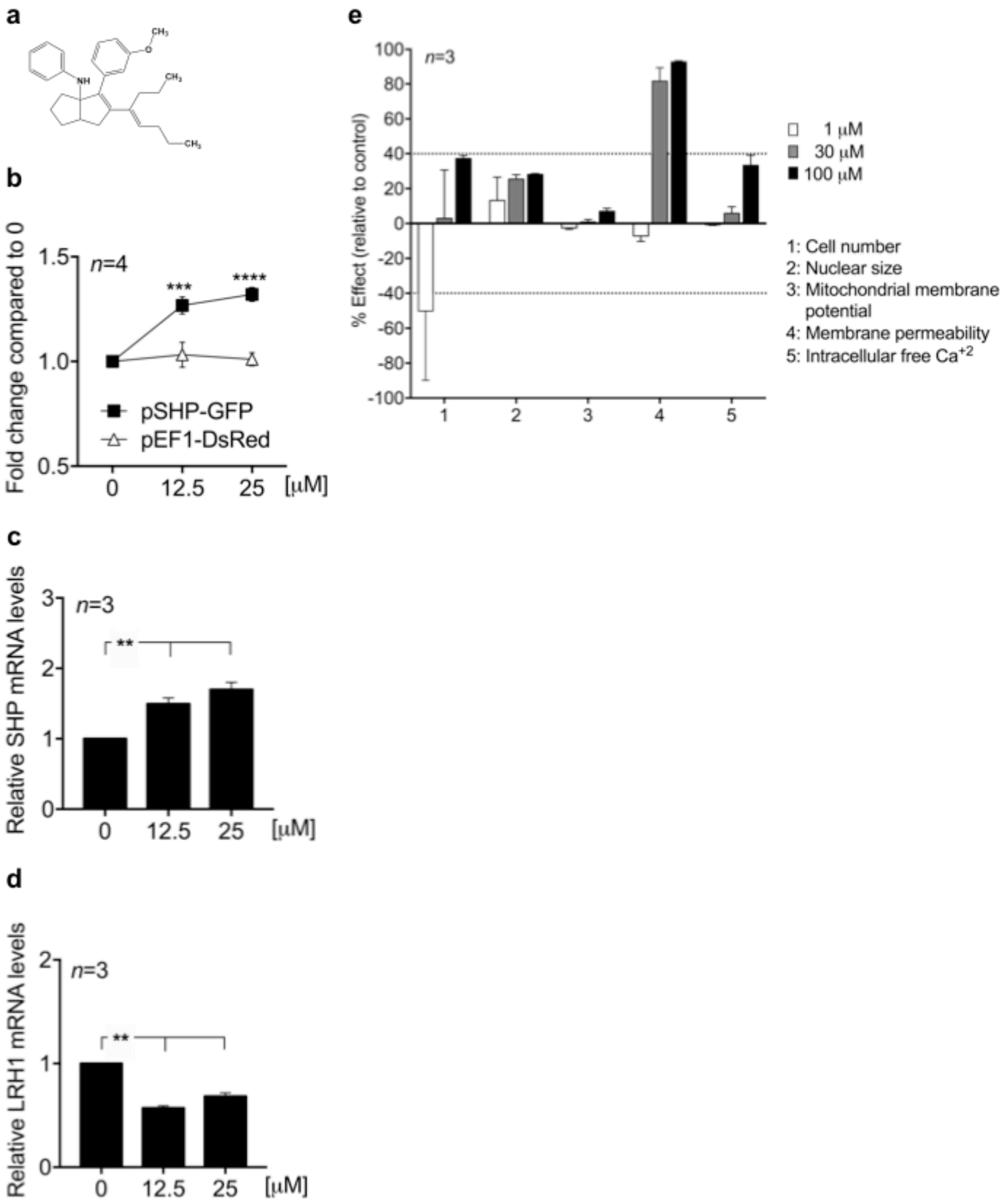

Supplementary Figure 1. BL001 enhances LRH-1 activity without adverse effects on cellular toxicity. (**a**) Chemical structure of BL001. (**b**) HepG2 cells stably transduced with both pSHP-GFP (LRH-1 target) and pEF1A-DsRed (control) constructs were incubated in the presence of increasing concentration of BL001. Cells were then analyzed by flow cytometry based on GFP and DsRed fluorescence. Results are presented as fold change in fluorescence as compared to control untreated cells (0 concentration). RNA was isolated from treated cells and transcript levels of endogenous SHP1 (**c**) and LRH-1 (**d**) were assessed by QT-PCR. (**e**) Cytotoxicity of BL001 on HepG2 cells was evaluated using the five endpoints assay, which simultaneously measures cell number, intracellular free calcium, nuclear size, membrane permeability and mitochondrial membrane potential. The dash line depicts the threshold of significant effect. Values are mean + or - s.e.m of (**b**) 3 and (**c-e**) 4 independent experiments each performed in triplicates. *p* values were assessed by two-way (**b**) or one-way ANOVA (**c** and **d**). ***p*<0.01 and ****p*<0.001.

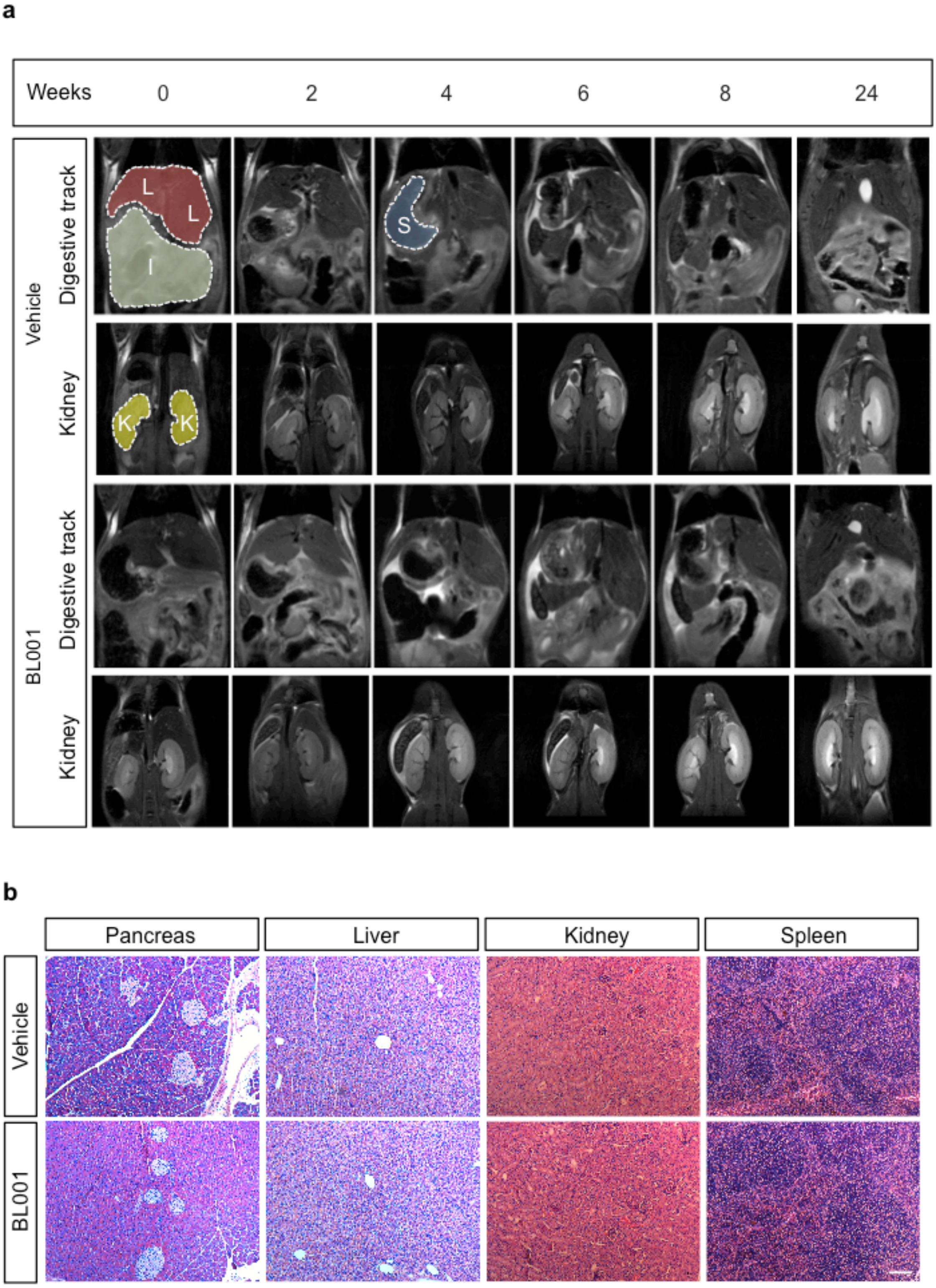

Supplementary Figure 2. Long-term administration of BL001 does not induce obvious macroscopic organ alterations. (**a**) Temporal MRI scans of either vehicle- or BL001-treated RIP-B7.1 mice reveal a control appearance of liver (L), intestine (I), spleen (S), and kidneys (K). (**b**) This was confirmed by the histological screening of HE-stained sections of the same organs. Scale bar: 100 μM.

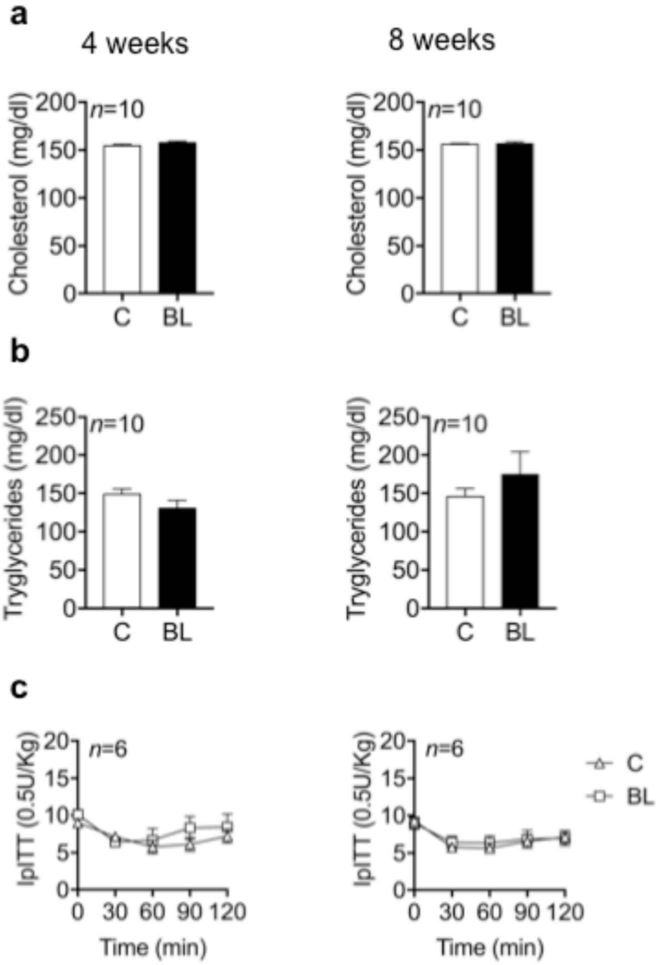

Supplementary Figure 3. BL001 does not alter the plasma lipid profile or the insulin sensitivity of RIP-B7.1 mice. The circulating levels of cholesterol (**a**) and triglycerides (**b**) were not altered after 4 and 8 wk of treatment with 10 mg/Kg b.w. BL001. Values are mean + s.e.m of 10 mice per group. (**c**) At the same time points, insulin tolerance tests (IpITT) revealed a control sensitivity of the BL001-treated mice. Values are means ± s.e.m of 6 mice per group. C, control; BL, BL001.

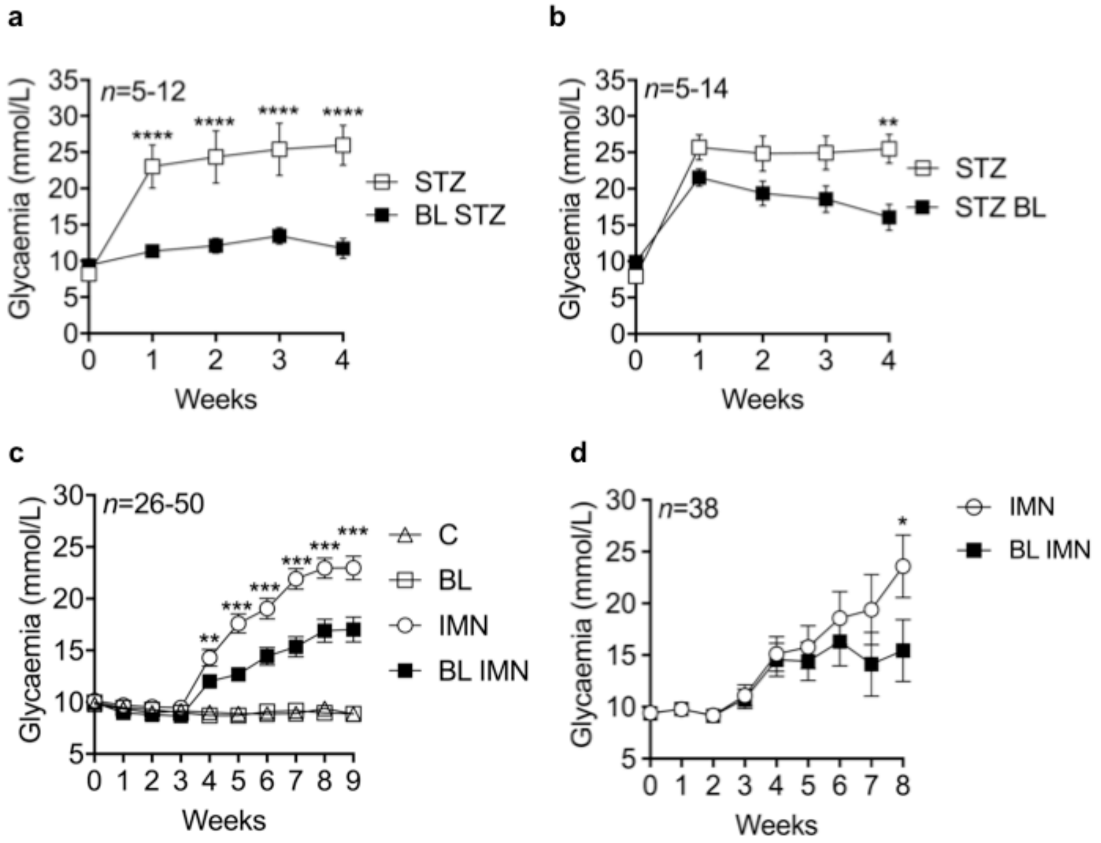

Supplementary Figure 4. BL001 blunts the development of diabetes in STZ-treated mice and RIP-B7.1 mice. Weekly glycemia of (**a**) C57BL/6 male mice treated or not with BL001 for 5 days prior to administration of a single dose of STZ (150 mg/Kg b.w.), (**b**) C57BL/6 male mice receiving STZ before initiation of BL001 treatment, (**c**) RIP-B7.1 mice of both genders pre-treated or not with BL001 for 5 days prior to immunization or not, and (**d**) RIP-B7.1 mice of both genders immunized at days 0 and 7 to induce a robust autoimmune response, and subsequently treated or not, with BL001 starting at week 1. Values are mean ± s.e.m. of (a) *n=5* (STZ), *n=12* (BL STZ); (b) *n=5* (STZ), *n=14* (BL STZ); (c) *n=26* (control, C), *n=26* (BL001, BL), *n=50*, (immunized, IMN); (d) *n=50*, (BL001 and immunized, BL IMN). *p* values were determined by two-way ANOVA. **p*<0.05, ***p*<0.01, ****p*<0.001 and *****p*<0.0001.

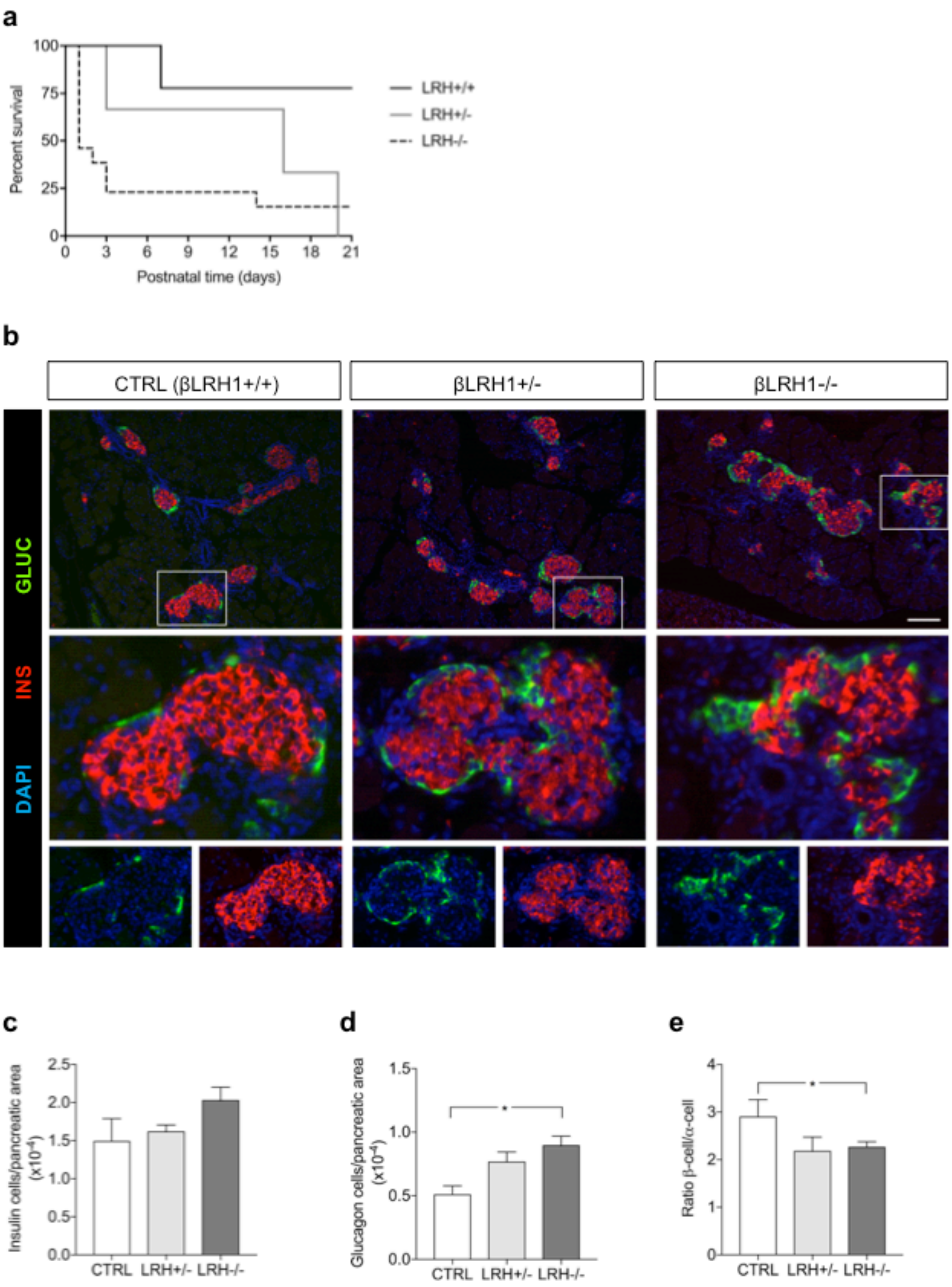

Supplementary Figure 5. Beta cell specific deletion of LRH-1 results in postnatal death. (**a**) Percent survival of pups prior to weaning. (**b**) Representative images of sections from control, βLRH1+/- and βLRH1+/- post-natal day 1 (P1) mice co-immunostained for insulin (INS, red) and glucagon (GLUC, green). Nuclei were stained with DAPI (blue). Lower images are enlargements of the white boxes as well as single INS and GLUC channels along with DAPI. Scale bar: 100 μM. Quantification of (**c**) beta and (**d**) alpha cell number in the various groups. (**e**) Ratio of beta to alpha cells. Values are also depicted as mean + s.e.m. (*n=4* mice). *p* values were determined by the one-way ANOVA. *$p<0.05$.

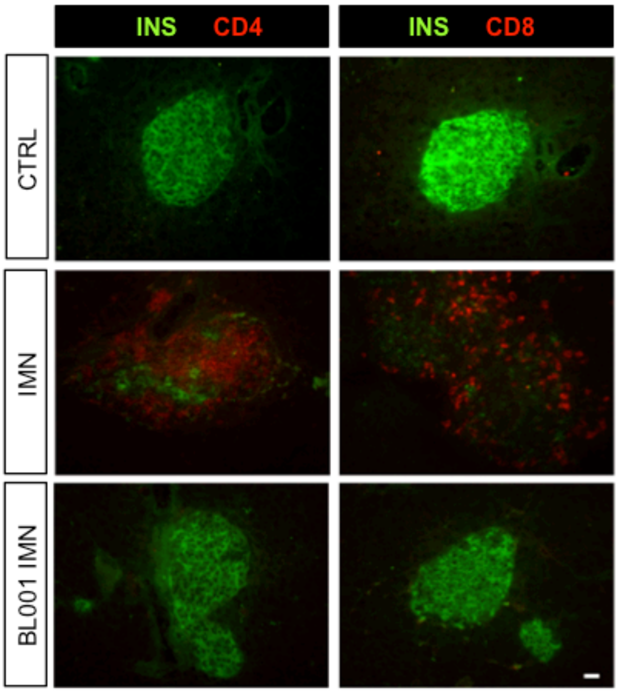

Supplementary Figure 6. BL001 prevents the islet infiltration by CD4^{+} and CD8+ T-cells. Immunostaining showed many CD4+ (left panels) and CD8+ lymphocytes (right panels) in islets of immunized (IMM) RIP-B7.1 mice (middle panels), but not in those of control (top panels) and of both IMM and BL001-treated mice (bottom panels). Insulin (INS, green); CD4 or CD8 (red). Scale bar: 25 µM.

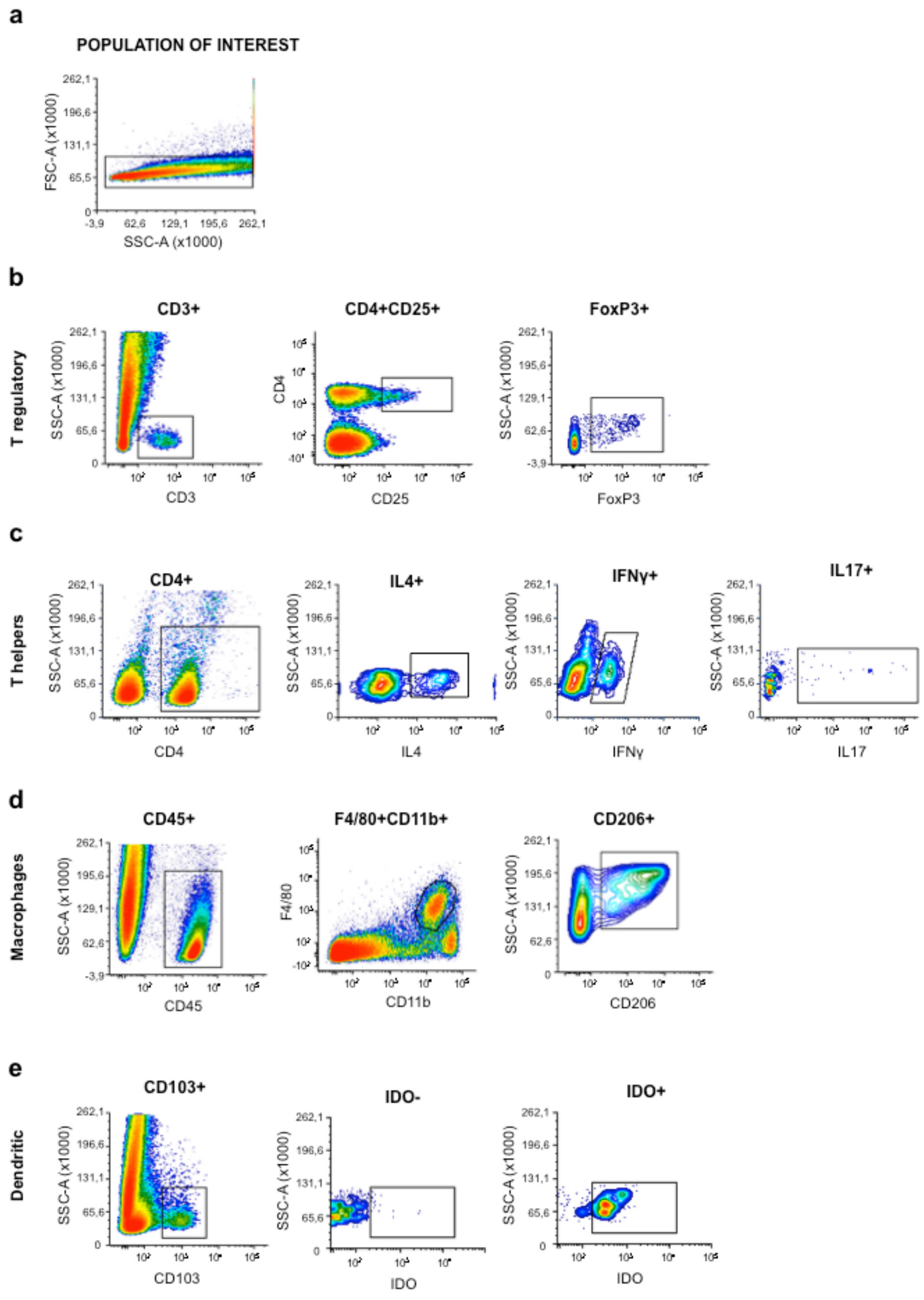

Supplementary Figure 7. Analysis of pancreatic immune cells. Flow cytometry gating strategy for sorting pancreatic immune cells. (**a**) Cells were gated on the basis of forward and side scatter. The square delineated subpopulation was then used to characterize the specific populations depicted in **Fig. 3a-g**. (**b** and **c**) T regulatory and helper cells were first gated on CD3^{+} and then analyzed for CD4, CD25 and foxP3 (Tregs, **Fig. 3a**) (**b**) or CD4, IL4 (Th2, **Fig. 3c**); CD4, IFN (Th1, **Fig. 3b**) and CD4, IL17 (Th17, **Fig. 3d**). (**d** and **e**) Macrophages and dendritic cells were initially gated for CD45 positivity. (**d**) Macrophages positive for both F4/80 and CD11b were further analyzed for CD206 expression (**Fig. 3f,g**). (**e**) Dendritic cells were gated for CD103 and then intracellular stained with IDO (**Fig. 3e**).

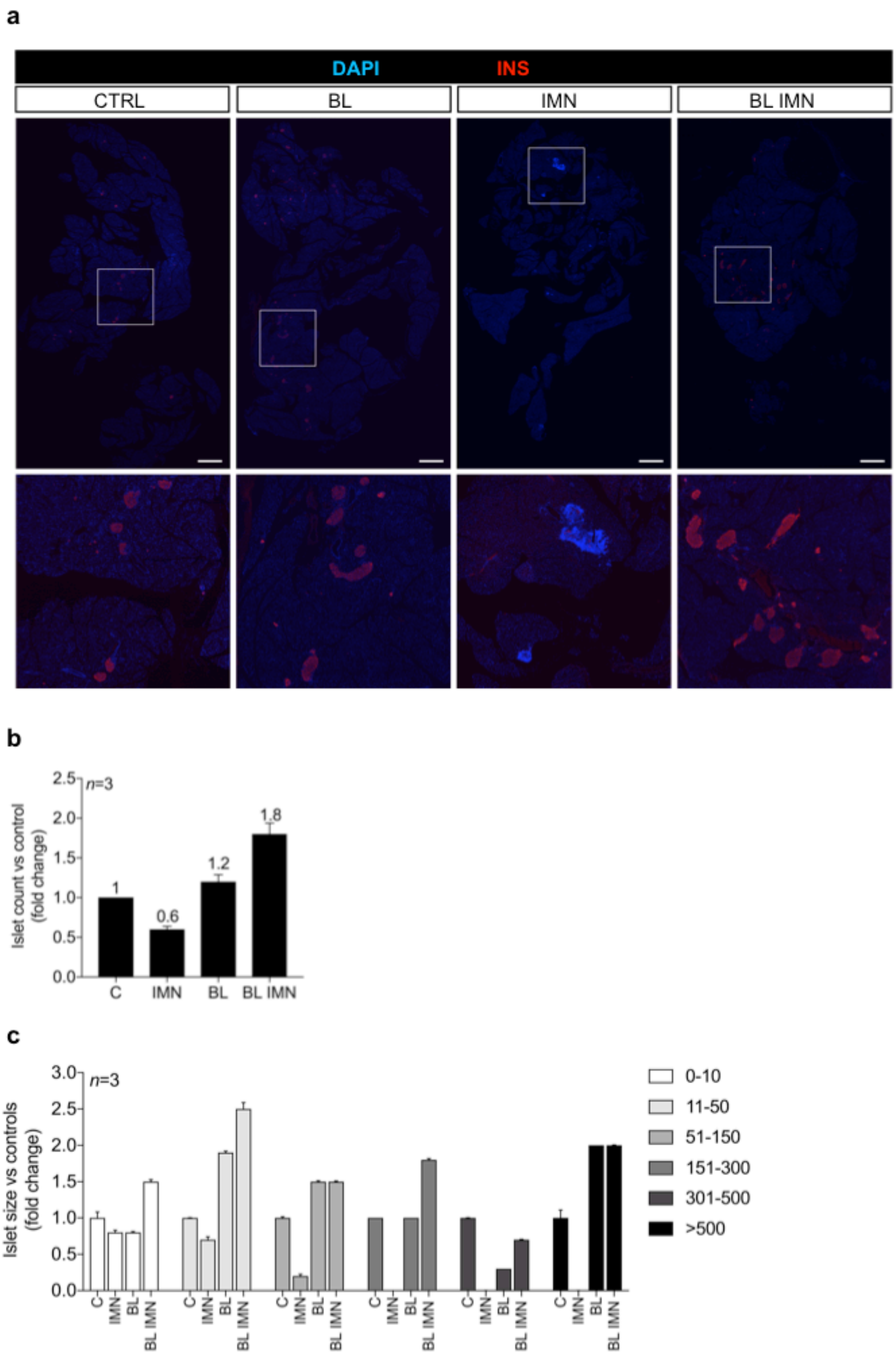

Supplementary Figure 8. BL001 increases the number of pancreatic islets. Immunostaining for insulin (red), and DAPI stain of nuclei (blue) revealed comparable islets in untreated (CTRL) and BL001-treated controls (BL001), but the near absence of islets in the RIP-B7.1 immunized animals (IMN). Bottom panels show enlargements of the pancreas areas boxed in top panels. The immunized mice which received the BL001 treatment (BL001 IMN) featured more numerous and larger islets than all other groups. Images representative of 3 pancreases per group. Scale Bar: 1000 µm. (**b**) Morphometry on the number (**c**) and size of islets. Values are mean + s.e.m. of *n=3* mice per group.

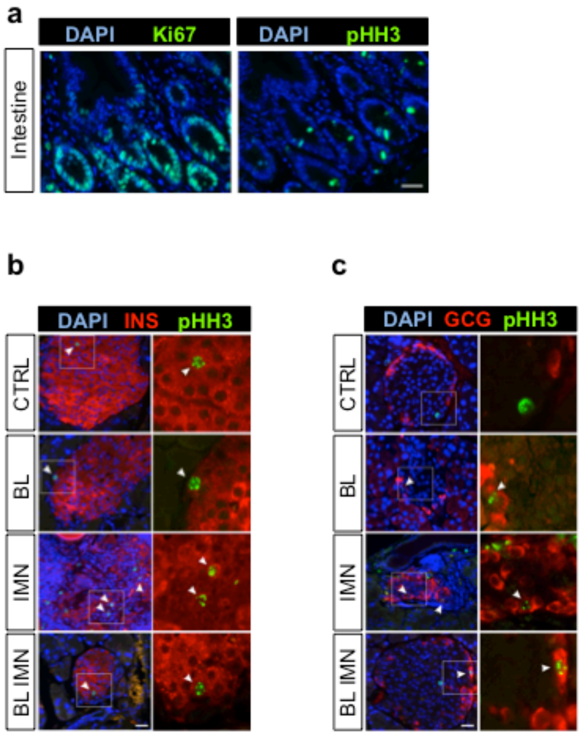

Supplementary Figure 9. BL001 does not stimulate beta and alpha cell proliferation. (**a**) Images of intestine immuhistochemical analysis using two independent proliferation markers: Ki67 (green, left panel) and the mitosis marker phospho-histone H3 (pHH3, green, right panel). Of note, the expression time domain of pHH3 is restricted as compared to Ki67 resulting in reduced number of positive. DAPI was used to stain nuclei. (**b-c**) Representative images of immunohistochemical analysis of pancreas section from control (CTRL), BL001 treated (BL), immunized (IMN) and BL001/immunized (BL IMN) RIP-B7.1 mice using anti-pHH3 (green, left panels) in combination with anti-insulin (red, **b**) or anti-glucagon (red, **c**) sera. Nuclei were stained with DAPI (blue). Insets in right panels are enlargments from preceding left panels (white squares). Scale bar: 25 μM (**a** and **b,c left panels**).

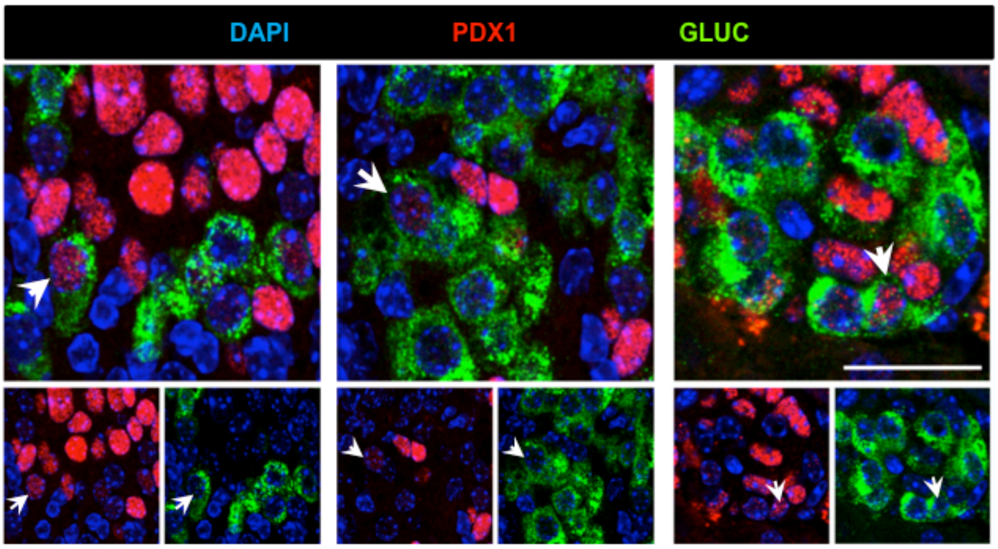

Supplementary Figure 10. Islets of immunized and BL001 treated RIP-B7.1 mice contain glucagon cells expressing PDX1. Three independent representative confocal images depicting co-immunostaining of PDX1 (red) and glucagon (green) in pancreatic islets of normoglycemic immunized BL001-treated mice (*n=3* pancreases). White arrows point to $GLUC^{+}/PDX1^{+}$ cells exhibiting various intensity of PDX1 staining. Lower images are single INS and GLUC channels along with DAPI. Scale bar: 25 μM.

a

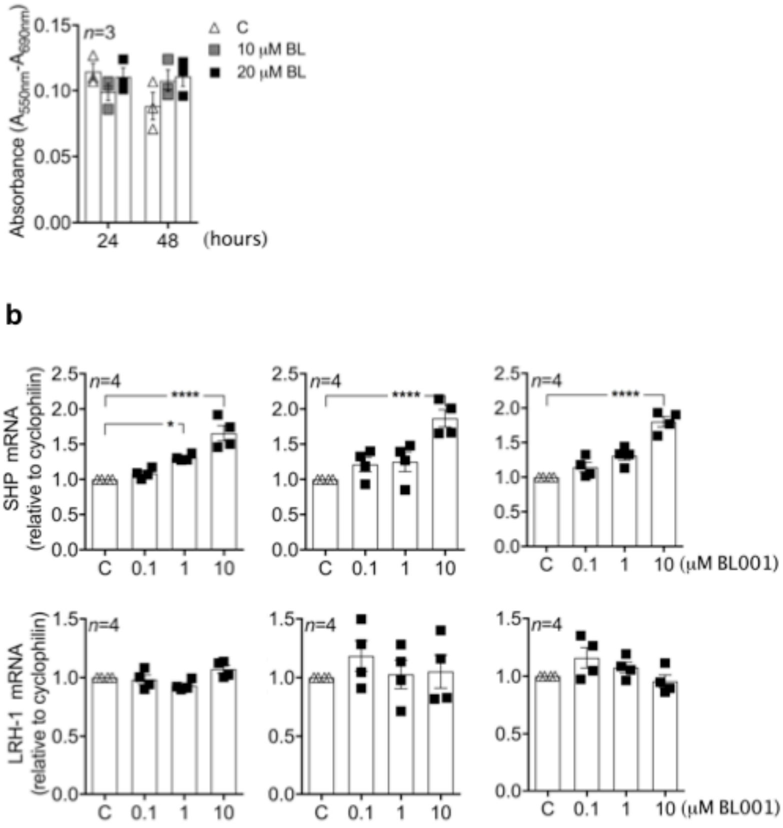

Supplementary Figure 11. BL001 induces SHP-1 expression human islets without altering viability. (**a**) MTT assay showed no cytotoxicity of a 24-48 h BL001 treatment of human islets (*n=3* independent islet preparations and each time point performed in triplicates). (**b**) QT-PCR analysis of SHP (upper panels) and LRH-1 (lower panels) mRNA levels in isolated human islets treated with increasing concentration of BL001 for 6, 12 and 48 h. C, control. (*n=4* independent islet preparations and each time point performed in triplicates). *p* values were determined by one-way ANOVA. *p<0.05 and ****p<0.0001.

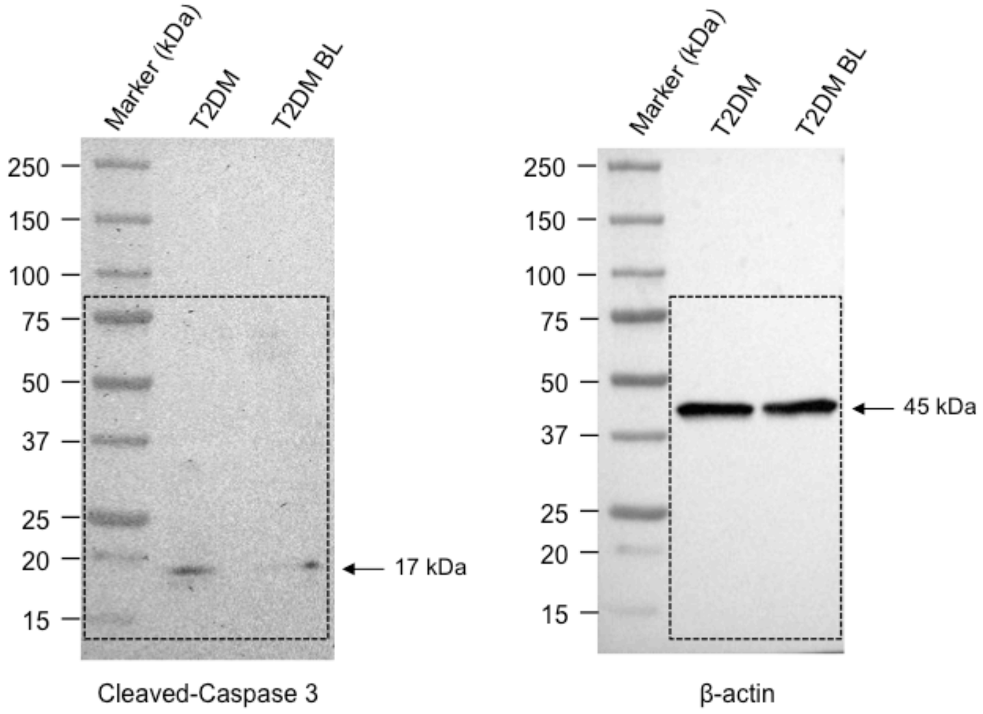

Supplementary Figure 12. Original blots for Figure 6i.

ND	Age (yrs)	Gender	BMI (Kg/m^2)	Cause of death
Donor #1	80	F	27.3	CVD
Donor #2	71	F	29.4	CVD
Donor #3	68	F	27.6	CVD
Donor #4	81	F	20.2	CVD
Donor #5	85	M	25.5	CVD
Donor #6	53	M	27.8	CVD
Donor #7	47	M	37.8	CVD
Donor #8	23	M	25.1	Trauma
Donor #9	59	M	30	Hemorrhage
Donor #10	36	F	21.5	Hemorrhage
Donor #11	58	F	25.8	Postanoxic encephalopathy
Donor #12	59	F	23.7	Trauma

Supplementary Table 1. Characteristics of non-diabetic donors. CVD; Cardio-vascular disease.

T2DM	Age (yrs)	Gender	BMI (Kg/m^2)	Cause of death
Donor #1	72	M	19.6	CVD
Donor #2	62	M	33.1	CVD
Donor #3	53	F	33.1	CVD
Donor #4	78	M	24.5	CVD
Donor #5	72	M	24.2	CVD
Donor #6	67	F	33.8	CVD
Donor #7	84	F	20.8	Trauma
Donor #8	81	M	26.4	CVD
Donor #9	79	F	21.5	CVD
Donor #10	72	M	27.7	CVD
Donor #11	52	M	34.9	CVD
Donor #12	69	M	28.4	Hemorrhage
Donor #13	58	F	22.5	CVD
Donor #14	64	M	26.1	CVD

Supplementary Table 2. Characteristics of donors with T2DM. CVD; cardio-vascular disease.

REAGENT	SOURCE	IDENTIFIER	DILUTION
Rabbit anti-insulin	Santa Cruz	sc-91168	1:200
Mouse anti-insulin	Sigma-Aldrich	I2018	1:250
Rabbit anti-glucagon	Cell Signalling	2760	1:200
Mouse anti-glucagon	Sigma-Aldrich	A944	1:150
Mouse anti Pdx-1	Hibridoma Bank	F109-D12-C	1:150
Rabbit anti-Ki67	Thermo Fisher Scientific	RM-9106	1:150
Goat anti-mouse IgG (H+L) Alexa Fluor 488	Thermo Fisher Scientific	A11001	1:800
Goat anti-mouse IgG (H+L) Alexa Fluor 568	Thermo Fisher Scientific	A11004	1:800
Rat anti-mouse CD4 FITC	BD pharmingen	553047	1:25
Rat IgG2a, k isotype Control RUO FITC	BD pharmingen	553929	1:25
Rat anti-mouse CD25 APC	BD pharmingen	557192	1:25
Rat IgG1, λ Isotype Control APC	BD pharmingen	550884	1:25
Rat anti-mouse FoxP3 PE	BD pharmingen	560408	1:10
Rat IgG2b, k Isotype Control PE	BD pharmingen	553989	1:10
Rat anti-mouse IL4 alexa fluor 647	BD pharmingen	557739	1:10
Rat IgG1, k Isotype Control Alexa fluor 647	BD pharmingen	557731	1:10
Rat anti-mouse IFNg PE-Cy7	BD pharmingen	557649	1:10
Rat IgG1, k Isotype Control PE-cy7	BD pharmingen	557645	1:10
Rat anti-mouse IL17 alexa fluor 647	BD pharmingen	560184	1:10
Rat anti-mouse I-A/I-E PE	BD pharmingen	562010	1:25
Rat anti-mouse CD11b FITC	BD pharmingen	557396	1:25
Rat anti-mouse CD8 alpha	Bio-rad	MCA2694	1:100
Rat anti-mouse CD4	Bio-rad	MCA1767	1:100
Anti-mouse CD3ε Brilliant Violet 421	Biolegend	100335	1:100
Armenian Hamster IgG Isotype Ctrl Brilliant Violet 421	Biolegend	400935	1:100
Anti-mouse CD45 Alexa Fluor 488	Biolegend	103121	1:100
Rat IgG2b, κ Isotype Ctrl Alexa Fluor 488	Biolegend	400625	1:100
Anti-mouse F4/80 APC	Biolegend	103121	1:50
Rat IgG2a, κ Isotype Ctrl APC	Biolegend	400511	1:50
Anti-mouse/human CD11b Brilliant Violet 421	Biolegend	101235	1:50
Rat IgG2b, κ Isotype Ctrl Brilliant Violet 421	Biolegend	400639	1:50
Anti-mouse CD206 (MMR) PE	Biolegend	141705	1:50
Rat IgG2a, κ Isotype Ctrl PE	Biolegend	400507	1:50
Anti-mouse CD103 PE	Biolegend	121405	1:100
Armenian Hamster IgG Isotype Ctrl PE	Biolegend	400907	1:100
Anti-IDO1 Alexa Fluor 647	Biolegend	654003	1:50
Mouse IgG1, κ Isotype Ctrl (ICFC) Alexa Fluor® 647	Biolegend	400135	1:50
Rabbit anti-mouse LRH1	Abcam	189876	1:100 (IF) 1:500(WB)
Anti-rabbit cleaved caspase-3	Cell Signaling Technology	9661	1:1000(WB)

Supplementary Table 3. Antibody list.
